# Impacts of Dietary Lysine and Crude Protein on Performance, Hepatic and Renal Functions, Biochemical Parameters, and Histomorphology of Small Intestine, Liver, and Kidney in Broiler Chickens

**DOI:** 10.3390/vetsci10020098

**Published:** 2023-01-29

**Authors:** Mohamed A. Mousa, Ahamed S. Asman, Reham M. J. Ali, Ramy K. A. Sayed, Kamlah A. Majrashi, Khloud G. Fakiha, Rashed A. Alhotan, Shaimaa Selim

**Affiliations:** 1 Department of Nutrition and Clinical Nutrition, Faculty of Veterinary Medicine, Sohag University, Sohag 82425, Egypt; 2 Department of Biochemistry, Faculty of Veterinary Medicine, Sohag University, Sohag 82425, Egypt; 3 Department of Anatomy and Embryology, Faculty of Veterinary Medicine, Sohag University, Sohag 82425, Egypt; 4 Biological Sciences Department, College of Science & Arts, King Abdulaziz University, Rabigh 21911, Saudi Arabia; 5 Department of Biology, College of Science, University of Jeddah, Jeddah 21493, Saudi Arabia; 6 Department of Animal Production, College of Food and Agriculture Sciences, King Saud University, Riyadh 11451, Saudi Arabia; 7 Department of Nutrition and Clinical Nutrition, Faculty of Veterinary Medicine, Menoufia University, Shibin El-Kom 32514, Egypt

**Keywords:** crude protein, lysine, biochemical traits, histomorphology, digestive organs, kidney, broilers

## Abstract

**Simple Summary:**

Protein and amino acids are essential for several biological activities in poultry. Therefore, the present study aimed to investigate the effects of increasing dietary lysine levels by 10% or 20% with an adequate dietary crude protein content, and the effects of a reduction in dietary crude protein content by 1% or 2% with the recommended amino acid content, on the performance, blood biochemical constituents, and histomorphology of the duodenum, liver, and kidney in broiler chickens. Our findings suggested that reducing dietary crude protein levels to lower than the recommended level did not negatively affect performance and induced minimal influence on the blood metabolic indicators of health status and moderate alterations in the organs’ histomorphology. Furthermore, increasing the lysine content by 10% above the recommended level was beneficial in improving growth performance, health status, and the histomorphology of the studied organs.

**Abstract:**

The present study aimed to investigate the effects of increasing dietary lysine (Lys) levels with an adequate dietary crude protein (CP) content, as well as the effects of a reduction in dietary CP content with the recommended amino acid (AAs) level, on the performance, blood biochemical parameters, and histomorphology of the duodenum, liver, and kidney in broiler chickens. A total of 500 broiler chickens were randomly distributed into five dietary treatment groups, following a completely randomized design, where, at the beginning, the control group (C) was fed a diet containing the standard CP and Lys levels: 23% CP with 1.44% Lys during the starter period; 21.5% CP with 1.29% Lys during the growing period; and 19.5% CP with 1.16% Lys during the finishing period. The Lys content was increased by 10% above the recommended control basal requirements in the second group (Gr1) and by 20% in the third group (Gr2), while using the same recommended CP percentage as the C group. The fourth group (Gr3) had a 1% lower CP content and the fifth group had a 2% lower CP content than the C group, with the same recommended AA level as the C group. Increasing the Lys content in the Gr1 group improved the broilers’ weight gains (*p* < 0.05) during the starter, growing, and finishing periods. Decreasing dietary CP with the standard AA levels (Gr3 and Gr4) did not significantly affect (*p* > 0.05) the live weight gain, feed intake, or feed conversion ratio (FCR) of the broilers compared with those fed with the C diet. Blood total bilirubin, direct and indirect bilirubin, triglycerides, cholesterol, low-density lipoprotein (LDL), and very LDL were not different among the experimental groups. However, blood aspartate aminotransferase levels were increased (*p* < 0.05) in the Gr1 and Gr3 groups compared with the other treatment groups. All dietary treatments decreased the serum creatinine levels (*p* < 0.05) compared with the C group. The Gr2 broilers had greater serum total protein and globulin (*p* < 0.05) than those receiving the other treatments. Increasing dietary Lys levels resulted in a significant improvement in duodenum villus height and width (*p* < 0.05), while the low-CP diets resulted in shorter villi length and width, along with degenerated areas and lymphocytic infiltration. Low dietary CP content induced hepatocyte disorganization and moderate degeneration, along with vacuolated hepatic cells, excessive connective tissue, and lymphocytic infiltration. The cortical regions of the kidney exhibited obvious alterations in the Gr3 and Gr4 groups and large interstitial spaces were found between tubules. Renal tubules in the Gr3 and Gr4 groups were smaller in size and some of these tubules were atrophied. In conclusion, reducing dietary CP levels to 1% or 2% lower than the recommended level did not negatively affect growth performance, inducing minimal influence on the blood metabolic indicators of health status, and resulting in moderate alterations to the histomorphology of the duodenum, liver, and kidney. Furthermore, increasing the Lys content by 10% above the recommended level improved the growth performance, health status, and histomorphology of the duodenum, liver, and kidney in broiler chickens.

## 1. Introduction

Over the past decade, poultry production has developed rapidly in tropical and subtropical regions, and it is predicted to maintain robust progress in the future. However, raw feed ingredients, particularly cereal grains and protein sources, are imported, causing an increase in the cost of these feed ingredients worldwide [1]. The price of poultry feed may be lessened with substitute diet formulations. Protein and amino acids (AA) are essential for several biological activities in poultry. AAs are critical to supporting broilers with rapid growth rates [2], and indeed some indispensable AAs are more important than others [3]. It has been documented that methionine, lysine (Lys), and threonine are the most limited AAs in corn-soybean meal-based diets [3,4,5]. Lys plays a major role in protein synthesis, muscle growth, the synthesis of cytokines, and the proliferation of lymphocytes, and thus in maximizing the immune response against infection [3,6,7]. Dietary Lys was reported to have beneficial impacts on the weight gain, feed consumption, feed efficiency, and meat yield of broiler chickens [8,9,10,11,12,13]. Therefore, the development of relevant information on AA content, particularly Lys, for broilers is necessary [5].

The remarkable increase in growth rate and muscle yield accomplished by the modern breeds selected for extraordinary meat production certainly implies that the demand for Lys supply exceeds that recommended for past strains. Consequently, commercial broiler diets are presently formulated with a higher Lys content than that recommended by the NRC [14] (1.10% total Lys from 0 to 21 days of age and 1.00% total Lys from 21 to 42 days of age). Previous research [15,16] has revealed that the Lys requirements of modern broilers are greater (1.001% and 0.995% digestible Lys from 28 to 42 days of age) than those previously suggested by the NRC [14]. Yang et al. [17] reported that broilers achieved faster weight gain and muscle yield when fed a relatively higher Lys content compared with those fed a low-Lys diet. Furthermore, Ishii et al. [18] observed that Lys supplementation above the NRC requirement (150%; 1.5% Lys) during the finishing period augmented the performance of broilers and they suggested that a high Lys diet may be a practical approach in the poultry industry. In support of this, Tian et al. [9] recorded that a reduction in dietary Lys (0.50% and 0.70%) diminished performance and fat deposition by downregulating the expression of lipogenic genes. Lys has attained significant importance in the demonstration of essential AA requirements due to its ratio, which is the basis of the ideal protein concept [19], and diet formulation that depends on an ideal protein concept being an effective approach to enhancing nitrogen utilization and minimizing nitrogen excretion. Therefore, determining the accurate Lys level is of the utmost importance in the field of poultry production [19,20] and supplemental Lys should be regularly evaluated as its recommended level can vary to satisfy production performance and economic requirements.

Another reasonable strategy that has been developed in poultry production as a potential alternative to feeding broilers with a high crude protein (CP) diet is a reduced CP diet with an adequate AA content. The key reasons for developing reduced-CP broiler diets are to decrease feed costs and to minimize nitrogen excretion, thus reducing environmental pollution [21,22]. Furthermore, decreases in nitrogen loss diminish the prevalence of footpad lesions and enhance bird welfare [21,22]. By balancing essential AAs, it appears possible to decrease dietary CP content by up to 3% without affecting the performance, feed efficiency, or carcass yield of broiler chickens [22,23,24]. However, more substantial reductions commonly compromise the FCR, with an associated enhancement in fat deposition [22,24]. Shao et al. [21] reported that reducing dietary CP content by 2% with adequate AA supply decreased nitrogen loss and footpad dermatitis without influencing the performance or meat quality of broilers.

The liver is considered the major site for protein synthesis and lysine catabolism [25,26]. Blood urea and uric acid can be used as practical response indicators to assess AA requirements or the efficiency of AA utilization in broiler chickens [27], and their levels were shown to increase as dietary nitrogen intake increased [27]. Previous studies have shown that excessive dietary CP or Lys can diminish hepatic physiological function, causing an accumulation of lipoprotein in the liver and liver damage [28,29]. Moreover, Xi et al. [30] demonstrated that high-CP diets were implicated in kidney and intestinal epithelial cell injuries as well as an imbalance of gut microbiota diversity. It appears necessary, therefore, to determine the histomorphology of the small intestine, liver, and kidney since histological alterations of these tissues can provide information on diet quality, nutrient metabolism, and the nutritional status of broiler chickens [25,26,29,30].

Even though several studies have been conducted to determine the recommended level of Lys content, research concerning the effect of increasing dietary Lys level has mainly been focused on the production performance and meat quality of the broilers. Moreover, little information is available on whether reducing the CP content by 1–2% can influence duodenal, hepatic, and renal histomorphology in Arbor Acres broiler chickens.

Therefore, this present trial aimed to investigate two strategies: increasing dietary Lys content by 10% or 20% above the breeder recommended level with an adequate dietary CP content; and reducing dietary CP content by 1% or 2% units, then examining their effects on performance, hepatic and renal functions, blood lipid profile, and the histomorphology of the duodenum, liver, and kidney in broiler chickens. In this study, we (1) hypothesized that increasing dietary Lys levels, relative to those recommended by breeder standards, could be effective in improving the performance, hepatic and renal functions, and histomorphology of the studied organs; and (2) examined whether decreasing dietary CP by 1% or 2% units would be effective in maintaining the performance, hepatic and renal functions, and histomorphology of the studied organs in Arbor Acres broilers.

## 2. Materials and Methods

This experiment was conducted in accordance with the guidelines of the Department of Nutrition and Clinical Nutrition, Faculty of Veterinary Medicine, Sohag University, Egypt. The ethical approval number was SOH. Vet. 022.1.18.

### 2.1. Experimental Housing, Management, and Design

The study was conducted at the Nutrition and Clinical Nutrition Research Center at the Faculty of Veterinary Medicine, Sohag University, Sohag, Egypt. The broilers were reared on a deep litter system with wood shavings. The trial was conducted from 10 to 42 days of age. The temperature was controlled and the lighting system was maintained at 23 h light and 1 h darkness. The diets were calculated according to the recommended nutrient requirements of the strain. The broiler chickens were fed a mash diet on an ad libitum basis and the health status of broiler chickens was monitored daily. All broilers were vaccinated against Newcastle disease at hatching and infectious bronchitis disease (IBD) at day 15. The broiler chickens were kept in an adaptation period for the first 10 days of age. All broiler chickens were individually weighed at 10 days of age to determine the initial body weight (BW), where the average BW was 365 ± 4.49 g. A total of 500 10-day-old male Arbor Acres Plus broiler chickens (https://eu.aviagen.com/assets/Tech_Center/AA_Broiler/ArborAcres-BroilerPerformanceObjectives2022-EN.pdf; accessed on 26 January 2023) were randomly divided into five groups (100 broilers per group with an average body weight of 365 ± 4.49 g), with each group subdivided into five replicates (20 broiler chickens per replicate). The control group (C) was fed a diet containing the standard CP and Lys levels: 23% CP with 1.44% Lys during the starter period; 21.5% CP with 1.29% Lys during the growing period; and 19.5% CP with 1.16% Lys during the finishing period. During the trial, the Lys content was increased above the recommended control basal requirements by 10% in the second group (Gr1) and by 20% in the third group (Gr2), while using the same recommended CP percentage as the C group. The fourth group (Gr3) had a 1% lower CP content and the fifth group had a 2% lower CP content, with an AA content similar to that of the C group. The broiler chickens had free access to feed and water during the trial. The diets were formulated to meet the nutrient requirements of broiler chickens, as recommended by Arbor Acres standards and as shown in Table 1, Table 2 and Table 3. For diet formulation, near-infrared reflectance spectroscopy, using a multi-purpose analyzer BRUKER (Hitachi, Inc., Tokyo, Japan), was performed to determine the ingredients for the AA contents [31]. The proximate chemical composition of the experimental diets was carried out in accordance with AOAC method [32].

### 2.2. Growth Performance

The body weight gain (BWG) and feed intake (FI) were recorded through the experimental periods (the first period lasted from 10 to 20 days of age, the second period lasted from 21 to 30 days of age, the third period lasted from 31 to 42 days of age, and the overall period lasted from 10 to 42 days of age). The calculated feed intake was the difference between feed supplied and refusal during each period. The FCR was calculated by dividing the feed intake by weight gain during the respective periods.

### 2.3. Blood Sampling

Blood samples (n = 5/replicate) were collected from the brachial wing vein at the end of the trial in tubes without anticoagulant for serum collection. Samples were centrifuged (3000 rpm for 5 min) at 20 °C until clear serum was obtained and stored at −20 °C until needed for further analyses. The serum samples were used to determine total protein (TP), albumin, globulin, urea, creatinine, aspartate aminotransferase (AST), alanine aminotransferase (ALT), total bilirubin, direct and indirect bilirubin, triglycerides, cholesterol, high-density lipoprotein (HDL), low-density lipoprotein (LDL), and very LDL. Biochemical analysis was performed according to the manufacturer’s recommendations (Bio-diagnostic Co., Cairo, Egypt) using a UV spectrophotometer (UV4802, Unico Co., Dayton, OH, USA).

### 2.4. Light Microscopic and Morphometric Analyses

For the histological investigations, samples (n = 5/replicate) from the liver, kidney, and small intestine (duodenum) were dissected from the experimental birds at the end of the experiment. The broiler chickens were selected (within the average BW of the group), after overnight fasting (8 h) and euthanized by cervical dislocation. The samples were washed with standard saline solution and fixed immediately in 10% neutral buffer formalin for 24 h. After proper fixation, the samples were washed under running tap water, dehydrated in ascending graded concentrations of ethanol, cleared with methyl benzoate, and embedded in paraffin wax. Sections (4–5 μm thickness) were cut, mounted on glass slides, and stained with Harris Hematoxylin and Eosin (H and E) stain for routine histological examination according to Bancroft and Stevens [33]. Staining was performed according to Bancroft’s theory and practice of histological techniques [34]. Sections were then examined using an OPTIKA B-293 microscope and digital images were acquired using an OPTIKA C-B10 camera and OPTIKA PRO View software. The length and width of the duodenal villi for a total of 9 obviously complete, full-sized villi showing no evidence of twisting or mechanical damage were determined from each sample. Morphometrical parameters were expressed in micrometers (µm) [35,36] using ImageJ software (National Institutes of Health, Bethesda, MD, USA) [37]. Four histologists (blinded to the groups) assessed the histological parameters.

### 2.5. Statistical Analysis

The normality of the collected data was determined via the Kolmogorov–Smirnov test and Levene’s test was used to ascertain the homogeneity of variance between treatments, and the assumption was accomplished at *p* > 0.05. The collected data were analyzed using one-way ANOVA in a completely randomized design using the IBM SPSS statistical package (version 22, SPSS Inc., Chicago, IL, USA) to determine the effect of the treatments. The means of all parameters were separated using Tukey’s test (*p* < 0.05). The experimental unit for growth performance data was a pen and the birds for other parameters.

## 3. Results

### 3.1. Growth Performance

There was a non-significant difference (*p* = 0.80) in the BW of broiler chickens at the start of the experiment (10 days of age). Increasing the Lys content by 10% over the recommended level resulted in a greater total live weight gain (*p* < 0.05) in broiler chickens compared with the other treatments (Table 4). Moreover, decreasing dietary CP with the recommended AA content did not result in high growth performance in the Gr3 and Gr4 groups (Table 4). The total body weight gain (BWG), cumulative feed intake (FI), and FCR recorded for the Gr3 and Gr4 groups were not significantly varied from those of the C group. Increasing dietary Lys content by 20% over the recommended level, even with the same CP percentage as the C group, increased the cumulative FI while diminishing the FCR (*p* < 0.05) of broilers during the whole experimental period (Table 4). The best cumulative FCR (*p* < 0.05) was recorded for the Gr1 broilers (10% Lys). However, the worst cumulative FCR (*p* < 0.05) was observed for the Gr2 group compared with the C and other treatment groups.

### 3.2. Biochemical Parameters

The data presented in Table 5 demonstrate the effects of different dietary treatments on the serum protein profiles of broiler chickens. All dietary treatments decreased the serum creatinine levels (*p* < 0.05) compared to the C group. However, the serum total protein and globulin concentrations were increased (*p* < 0.05) in all the studied groups compared to the C group. The Gr1, Gr2, and Gr4 broilers had lower serum urea contents (*p* < 0.05) than the C and Gr3 broilers. There was a non-significant difference in the serum albumin levels among the treatment groups.

The data shown in Table 6 demonstrate the effects of different dietary treatments on the hepatic and renal functions of broiler chickens. There was a non-significant difference in the serum alanine aminotransferase (ALT), total bilirubin, and direct and indirect bilirubin concentrations between the experimental groups and the C group. The Gr1 (10% increase in Lys) and Gr3 (1% lower CP with the recommended AA level) broilers had a greater serum aspartate aminotransferase (AST) content (*p* < 0.05) than the other experimental groups. The serum lipid profiles of broiler chickens fed different dietary treatments are shown in Table 7. Non-significant differences were noticed in cholesterol, triglycerides, or LDL between the treatment groups. Broiler chickens fed the Gr1 diet had higher serum HDL levels than those receiving the C diet.

### 3.3. Histomorphological Findings

#### 3.3.1. Liver

**Figure 1:**
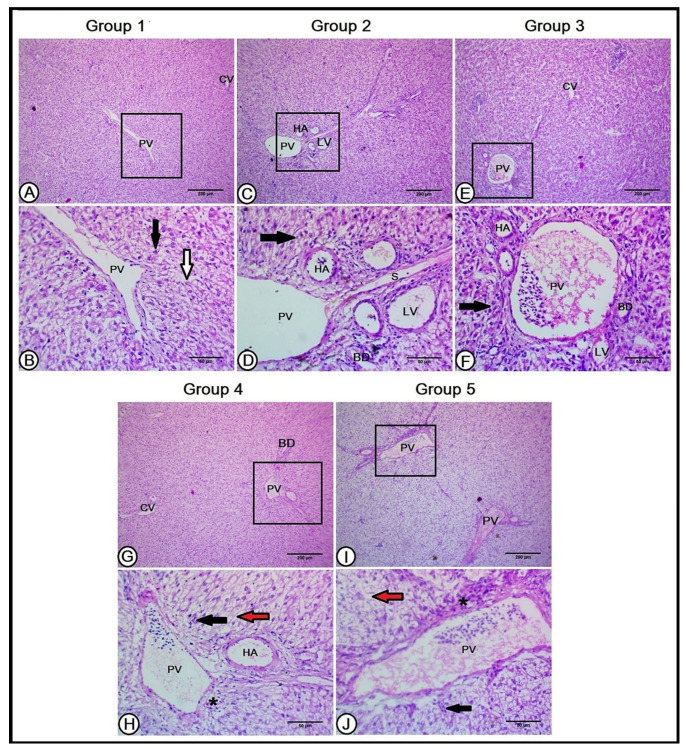
A histopathological analysis of the hepatic architecture in the experimental groups (**A**,**B**): the livers in the first group (Con) showed normal hepatic architecture. The central vein (CV) was in the center of each hepatic lobule and the hepatocytes (black arrow) were arranged in plates that radiate longitudinally outward from the central vein that was separated by a hepatic sinusoid (white arrow). The portal triad contained a branch of the portal vein (PV). The livers of the second and third groups (**C**–**F**, respectively) revealed a better organization of the hepatic cells (black arrows) and hepatic sinusoid (S) in between. The portal triad is composed of a large-sized branch of the portal vein (PV), with numerous branches of the hepatic artery (HA), and bile ducts (BD), in addition to lymphatic tissues (LV). The livers in the fourth and fifth groups (**G**–**J**, respectively) illustrate disorganized and degenerated hepatocytes (black arrows) around the central vein (CV), numerous variable-sized hepatic sinusoids (red arrows), excessive connective tissue, lymphocytic infiltration (black asterisks), fewer branches of the hepatic artery (HA), and bile duct (BD).

A histological analysis of the livers in the C group showed normal hepatic architecture, where the arrangement and structure of the lobules, central veins, and hepatic platelets were clear. The central vein was in the center of each hepatic lobule and the hepatocytes were arranged in plates that radiated longitudinally outward from the central vein, toward the edges of the lobules. The connective tissues separating the lobules from each other were unclear, except for those presented in the portal triad, which contained branches of the portal vein, hepatic artery, bile duct, and lymphatic vessels. The hepatic cells were arranged in hepatic laminae that were separated by a hepatic sinusoid, which received blood from the hepatic artery and portal vein and conducted to the central vein (Figure 1A,B).

The hepatic structure was improved in the Gr1 and Gr2 groups, where the hepatocyte arrangement was obvious, with fewer sinusoidal spaces. The portal triad exhibited a large-sized branch of the portal vein, with fewer connective tissue aggregations and numerous branches of the hepatic artery and bile ducts, in addition to lymphatic tissues (Figure 1C–F). Reducing the percentage of CP with the recommended AA level in the Gr3 and Gr4 groups induced the disorganization of hepatocytes with moderate degeneration and vacuolated hepatic cells. Numerous variable-sized hepatic sinusoids were observed, in addition to excessive connective tissue and lymphocytic infiltration found in the portal triad. Fewer branches of the hepatic artery and bile duct were depicted (Figure 1G–J).

#### 3.3.2. Kidney

**Figure 2:**
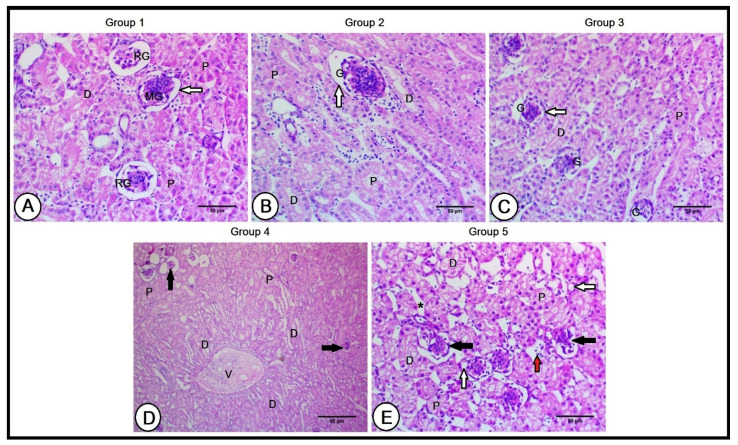
A histopathological analysis of the renal cortex in the experimental groups (**A**): Renal cortex of the control group showing glomeruli of reptilian-type nephrons (RG) and glomeruli of mammalian nephrons (MG), and proximal (P) and distal (D) convoluted tubules. The glomerulus was surrounded by thin glomerular basement membranes (black arrow). The renal cortex of the second (**B**) and third (**C**) groups revealed large-sized proximal (P) and distal (D) convoluted tubules. The glomeruli (G) increased in size in the second group; however, their size was slightly reduced in the third group. The glomeruli were surrounded by thin glomerular basement membranes. The renal cortex of the fourth group (**D**) shows small-sized glomeruli (black arrows) and a concentric arrangement of distal convoluted tubules (**D**) around the intralobular vein (V). The renal cortex of the fifth group (**E**), illustrating small-sized glomeruli, surrounded by irregularly disrupted basement membranes (black arrows), and large interstitial spaces (black asterisk) between small-sized proximal (P) and distal (D) convoluted tubules. Note the presence of atrophied tubules (white arrows) and few lymphocytic infiltrations (red arrows).

A histological examination of the kidneys in the C group showed a normal renal structure. The kidney comprises two zones: the cortex and the medulla. The cortical region of the studied birds’ kidneys was intensely stained, comprising glomeruli, and proximal and distal convoluted tubules. The two main types of nephrons are reptilian-type nephrons with smaller glomeruli and mammalian nephrons that possess loops of Henle. The kidneys of the C group showed a high density of reptilian-type nephrons and a lower density of mammalian-type nephrons distributed throughout the cortical region. The glomerulus was surrounded by thin glomerular basement membranes (Figure 2A).

The renal cortical structure preserved this architecture in the Gr1 and Gr2 groups, resulting in considerable improvements, including large-sized proximal and distal convoluted tubules. The glomeruli increased in size in the Gr1 group; however, their size was slightly reduced in the Gr2 group and surrounded by thin glomerular basement membranes in both groups (Figure 2B,C). The cortical regions exhibited obvious alterations in the Gr3 and Gr4 groups; the glomeruli were smaller in size, and their basement membranes were irregularly disrupted (Figure 2E). A concentric arrangement of distal convoluted tubules around the intralobular vein was observed (Figure 2D). Large interstitial spaces were found between tubules, which were smaller in size, and some of these tubules were atrophied. Moreover, few lymphocytic infiltrations were depicted (Figure 2E).

#### 3.3.3. Small Intestine (Duodenum)

**Figure 3:**
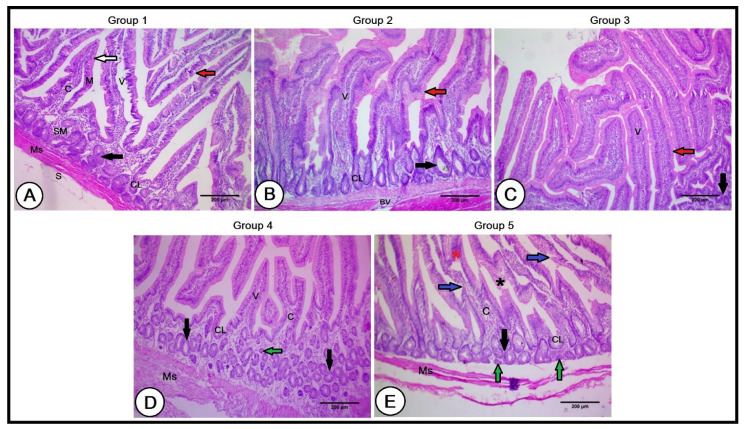
A histopathological analysis of the small intestines in the experimental groups (**A**): Duodenum of the control group showing mucosa (M), submucosa (SM), muscularis (Ms), and serosa (S) tunics. The lamina epithelialis consisted of simple columnar epithelial cells (white arrows) and goblet cells scattered among them (red arrows). The intestinal villi consisted of connective tissue core (C) and exhibited intestinal glands (crypts of Lieberkühn, CL). The submucosa revealed submucosal glands (black arrows). (**B**,**C**): Intestinal wall of the second and third groups, respectively, revealing longer and wider intestinal villi (V), numerous goblet cells (red arrows), deeper and larger crypts of Lieberkühn (CL), and large-sized submucosal glands in the second group, with the beginning of their size reduction in the third group (black arrows). (**D**): Duodenal wall of the fourth group showing reduced intestinal villi (V) packed with a less connective tissue core (C). The intestinal glands (CL) were smaller and the submucosa glands were numerous and small in their size (black arrows). (**E**): Duodenal wall of the fifth group illustrating short intestinal villi with irregular lamina epithelialis (blue arrows), degenerated areas (black asterisk), lymphocytic infiltration (red asterisk), poor connective tissue cores (C), and smallest and shortest intestinal glands (CL). Note the presence of numerous small-sized submucosal glands (black arrows), together with some atrophied ones (green arrows), and a narrow muscular tunic (Ms).

A histological analysis of the small intestines in the C group revealed normal intestinal wall architecture, formed of the mucosa, submucosa, muscularis, and serosa tunics. The mucosa consisted of a lamina epithelialis of simple columnar epithelial cells and goblet cells scattered among them. The lamina propria was presented by a layer of loose connective tissue containing collagenous, elastic, and reticular fibers, in addition to lymph and blood vessels, nerve fibers, and intestinal glands (crypts of Lieberkühn). The intestinal villi were 556.34 ± 85.57 µm in length and 151.77 ± 19.86 µm in width, consisting of a connective tissue core derived from the lamina propria, covered with a cap of simple columnar epithelium and goblet cells. The submucosa was composed of loose connective tissue containing blood and lymph vessels, nerve plexus, and submucosal glands. The muscularis tunic of the smooth muscle fibers was well arranged in inner circular and outer longitudinal layers (Figure 3A).

The intestinal structure was improved in the Gr1 and Gr2 experimental groups, with longer and wider intestinal villi; however, this increment was higher in the Gr1 group than in the Gr2 group (676.12 ± 49.60 µm and 611.89 ± 79.96 µm in length in the second and third groups, respectively; 186.27 ± 22.48 µm and 156.2 ± 29.84 µm in width in the second and third groups, respectively). Moreover, the lamina epithelialis contained numerous goblet cells scattered among simple columnar epithelial cells. The villi were compacted with dense connective tissue cores, and the crypts of Lieberkühn were deeper and larger. Furthermore, the submucosal glands were larger in size (Figure 3B,C).

In the Gr3 group, the intestinal villi revealed a reduction in their length (406.89 ± 65.57 µm), and width (100.72 ± 16.34 µm), and were packed with a less connective tissue core. The intestinal glands were smaller and the submucosal glands were numerous and small in their size (Figure 3D). In the Gr4 group, the lamina epithelialis of the intestinal villi depicted irregularity in some regions, with the presence of degenerated areas and lymphocytic infiltrations. The intestinal villi had poor connective tissue cores and the smallest and shortest intestinal glands. The villi length and width showed a marked decline (365.48 ± 94.59 µm and 81.68 ± 11.49 µm, respectively). Numerous small-sized submucosal glands, together with some atrophied ones, were also observed. The muscular tunic was narrower, with wide interstitial spaces (Figure 3E).

## 4. Discussion

Even though Lys is not the first indispensable AA in corn-soybean meal-based diets, it is feasibly the most essential AA for broiler chickens, as it is considered the standard AA for the ideal protein concept in feed formulation [38]. Dietary supplementation with AAs, including Lys, permits nutritionists to adjust requirements and thus augment performance while decreasing the demand for protein sources [3,6,7]. Identifying the optimal Lys requirement is of economic value because consuming diets either deficient in Lys or with excess Lys causes poor growth rates and/or rising feed costs [9]. On the other hand, reducing the CP content in broiler diets has mainly been developed to decrease feed costs and minimize nitrogen excretion, thus reducing environmental pollution [21,22]. Therefore, in the present study, we investigated the effect of increasing the dietary Lys level by 10% or 20% above the breeder-recommended level with an adequate dietary CP content, as well as a reduction in the dietary CP content by 1 or 2% units with the recommended AA content, on the performance, hepatic and renal functions, blood lipid profile, and histomorphology of the duodenum, liver, and kidney in broiler chickens.

During the starter period, the growth performance of the broilers was significantly improved in the Gr2 group compared with the C group. These results could be attributed to the increase in available indispensable AA Lys, which plays a key role in muscle growth, especially breast muscle, where it is important in muscle protein synthesis [3,6,7]. Lys is essential in the biochemical reactions necessary for the growth of broiler chickens during the early stage of life [9]. Further, during the first two weeks of age, there are smaller digestive enzyme concentrations, crucial for the liberation of AA from feed [39], and the crystalline AAs added to broiler diets are more digestible compared with bounded AAs [40]. During the growing period, increasing the Lys content from 1.29% (C) to 1.4% (Gr1), with the standard requirement of CP in the diet, significantly improved the FCR and BWG of the broilers. However, broiler chickens fed a diet containing 1.53% Lys (a 20% increase) with the standard CP requirement (Gr2) did not perform as well as the control broilers, suggesting that a dietary inclusion threshold had been exceeded during the growing period and that a 10% increase in Lys content above the breeder recommendation was beneficial during this period of life. Bouyeh [41] reported a reduction in weight gain when feeding broiler chickens with a Lys content that was 40% greater than the NRC recommendation (1.54% and 1.40% during the starter and growing periods, respectively). Recently, Jespersen et al. [42] observed that doubling the Lys content at 2.13% and 1.95% in the starter and grower diets compared to the NRC recommendation slowed the growth rate of broiler chickens. They attributed this decrease in the growth performance of those birds to a deficiency of arginine [42]. Tian et al. [9] observed that excessive supplementation with Lys depressed the body weight and gain of broilers. An et al. [43] observed that growth in 21- to 28-day-old broiler chickens improved as dietary standardized ileal digestible Lys rose from 7.5 g/kg to 12.5 g/kg, approximating that the Lys requirements for the greatest body gain and feed efficiency were 11.6 g/kg and 12.1 g/kg, respectively. Moreover, Ishii et al. [18] found that Lys supplementation above the NRC requirement (150% NRC; 1.5% Lys) during the finishing period (21 to 38 days of age) augmented the performance of broilers, suggesting that a high-Lys diet may be a practical approach in the poultry industry. In the current study, we suggested that the decreased growth performance of the Gr2 broilers during the growing and finishing periods may have been due to excess Lys intake, which might lower the utilization of other AAs. Moreover, the decreased FCR in the Gr2 broilers may be due to the increased FI, which was unexpected. The mechanism underlying the increased FI with reduced BWG due to excess dietary Lys concentration (20% above the recommended level) was not clear and warrants further research.

A decrease in CP content by 1% or 2% CP units with standard AA contents did not significantly affect the overall growth performance compared with the control group. Our findings are consistent with Shazali et al. [7], Shao et al. [21], and Van Harn et al. [22], who observed that low-CP feeding programs (2%, 1–3%, and 1–2% fewer CP units, respectively) with balanced AAs did not affect the BWG, FI, or mortality of broiler chickens. Moreover, Chrystal et al. [24] reported that reduced dietary CP from 21% to 18% did not negatively impact the growth performance of broilers, while an additional decrease in CP from 18% to 16.5% compromised the FCR, even though the concentrations of essential AAs were adequate. Our results indicated that a corn-soybean basal diet with 1–2% fewer CP units and adequate AA content could meet the protein requirement of Arbor Acres broilers. Indeed, offering reduced-protein diets to broiler chickens lessens the use of soybean meal and can be an effective approach to enhancing intestinal health and animal welfare, as well as decreasing ammonia emissions [21,22].

The duodenal histomorphology of the control broilers was consistent with previous studies [44,45]. Earlier studies have reported that as villus length and width improve, both the digestive and absorptive activities of the small intestine augment with a resultant increase in the absorptive surface area, the expression of brush border enzymes, nutrient transport, and body weight [44,45]. The improvement in the growth performance of the broiler chickens fed the Gr1 diet was supported by duodenal histomorphology which revealed longer and wider duodenal villi in the Gr1 broilers than in the control group, whereas the other treatment groups showed shorter villi and slowed growth rates. The consumption of the Gr2 diet resulted in a reduction in the length, width, and, thus, the surface area of villi compared with the Gr1 diet, which may be due to the suppression of beneficial intestinal microbiota [44], therefore resulting in a slowed growth performance. Previous reports have shown pathological lesions and alterations in the intestinal villi based on the type of diet in different animal species. Nonetheless, few reports have determined the relationship between dietary CP levels in broilers and intestinal development. Incharoen et al. [46] suggested that protein is critical among other macronutrients for the development of the intestinal villi and epithelial cells. In the current study, a decreased CP percentage by 1 and 2 units resulted in shorter villi with fewer intestinal glands, degenerated areas, and lymphocytic infiltrations, and these changes in the duodenum were markedly pronounced in the Gr4 group (−2% CP). The current study implies that these alterations in the duodenal histomorphology of the Gr4 broiler chickens due to being fed a low-CP diet may have slowed the growth performance of the broilers. A reduction in villus height may result in poor nutrient absorption and performance. In support of these findings, the Gr4 broilers had numerically lower body gain, feed intake, and feed efficiency compared with the control group. The detrimental effect of low-CP diets on intestinal morphology may be due to the lower content of non-essential AAs which efficiently sustains the intestinal epithelial layer [47]. Recently, Kuritza et al. [48] observed a reduction in performance and diminished intestinal histological measurements, including villus, crypt, and goblet cells, in broiler chickens fed with low-CP diets (1–3 fewer CP percentage units).

Blood biochemical analysis is a standard practical way to assess the health status and to reveal the nutritional status of poultry. Liver and kidney histomorphology are important approaches because their histological alterations can provide information on diet quality, nutrient metabolism, and the nutritional status of broiler chickens [25,26,29,30]. In the present study, increasing Lys or decreasing dietary CP content resulted in significant decreases in serum creatinine and urea levels compared with the control. Blood urea can be used as a vital indicator to assess AA requirements or AA utilization efficiency in broiler chickens [27]. The serum urea reveals AA metabolism, with higher AA catabolism associated with greater urea concentration [27]. Wang et al. [49] observed that serum urea concentration was not significantly affected by dietary CP reduction by up to 4.5%, but it was raised with a decrease in dietary CP by 6%. A reduction in serum urea content was shown with a reduction in dietary CP (by up to 4.5% or 6%) [50,51] or with increasing dietary Lys (150% NRC; 1.50% Lys) [18]. They suggested that the decrease in serum urea could be associated with lower ingestion, with the subsequent decrease in the catabolism of AA [49,50,51]. The decrease in blood urea level in the current study may suggest enhanced protein utilization and synthesis in the broiler chickens fed a diet with more Lys or those fed a low-CP diet with the recommended AA content. Renal damage and abnormal catabolic activity are known to be accompanied by elevated blood urea, uric acid, and creatinine concentrations [52]. Despite the cortical regions of the kidney exhibiting obvious moderate alterations and the presence of some atrophied tubules in the Gr3 and Gr4 groups, the kidneys of these broilers were still functioning without showing renal damage and the changes in the kidney structure did not affect the health of the broiler chickens so much. Little information is available in the literature concerning the effect of either reducing dietary CP or increasing dietary Lys on the kidney histomorphology of broiler chickens to compare with the findings reported herein.

The liver is considered the major site for protein synthesis and lysine catabolism [25,26]. The hepatic structure was improved in the Gr1 and Gr2 groups. Where the hepatocyte arrangement was obvious, the portal triad exhibited a large-sized branch of the portal vein, with fewer connective tissue aggregations and numerous branches of the hepatic artery and bile ducts, in addition to lymphatic tissues. In support of this, serum ALT, AST, triglycerides, cholesterol, LDL, VLDL, total bilirubin, and direct and indirect bilirubin contents remained unchanged in response to increasing dietary Lys. Blood albumin and total protein concentrations are trustworthy indicators of hepatic function and, collectively with blood AST and ALT levels and blood lipid profile, are closely related to the extent of hepatic lipidosis [53,54]. Excessive dietary L-lysine·H_2_SO_4_ was reported to diminish the hepatic physiological function, causing the deposition of lipoprotein in the liver and liver damage [29]. Accordingly, our findings implied that the status of lipid metabolism and liver physiological function were not impaired, so it could be that increasing Lys by 10% has favorable health impacts on broiler chickens. On the other hand, serum AST activity was elevated with decreasing CP levels and this elevation was associated with the disorganization of hepatocytes, moderate degeneration of hepatic cells, vacuolated hepatic cells, excessive connective tissue, and lymphocytic infiltration. Our results indicated that the liver function status might be, to some extent, disrupted by decreasing the dietary CP content [53]. Consistent with our findings, Zhang et al. [53] reported an increase in serum ALT and AST activities with decreasing CP levels. In the current study, there were non-significant differences in serum bilirubin, indirect and direct bilirubin, cholesterol, triglycerides, and LDL contents between broilers fed the reduced-CP diets and those fed the C diet, implying that lipid metabolism and hepatic functions were not detrimentally affected by decreasing the dietary CP (1% or 2%) contents in Arbor Acres broiler chickens.

## 5. Conclusions

In the present study, we observed that supplementation with Lys at 10% above the breeder-recommended level improved the growth performance, metabolic indicators of health status, and histomorphology of the duodenum, liver, and kidney of the broiler chickens to a greater extent than that of the control group. Conversely, Lys supplementation at 20% above the recommended level improved the growth performance of the broilers during the period from 10 to 21 days of age only, while inducing negative impacts on the growth performance during the growing and finishing periods. Furthermore, decreasing CP by 1% or 2% below the recommended level did not significantly affect the growth performance and induced minimal influence on the blood metabolic indicators of health status. The low-CP diets induced shorter duodenal villi lengths and widths along with degenerated areas and lymphocytic infiltration. Low CP content with recommended AA content induced the disorganization of hepatocytes and moderate degeneration, along with vacuolated hepatic cells, excessive connective tissue, and lymphocytic infiltration. The cortical regions of the kidney exhibited obvious alterations in the Gr3 and Gr4 groups and large interstitial spaces were found between the tubules, which were smaller in size, and in some cases atrophied. In conclusion, despite the noticeable moderate alterations in the duodenum, kidney, and liver structures in broiler chickens fed low-CP (−2%) diets, the performance and metabolic indicators of health status were not detrimentally affected. Furthermore, increasing the Lys content by 10% above the breeder-recommended level may serve as a practical approach to improving the growth performance and health status of Arbor Acres broiler chickens.

## Figures and Tables

**Table 1:** The composition of the experimental diets and proximate chemical analysis during the starter period.

Groups	C	Gr1	Gr2	Gr3	Gr 4
CP%	Standard protein 23%			22% CP	21% CP
Lysine content	1.44% Lys	1.54% (110% from basal ration)	1.68 % (120% from basal ration)	1.44%	1.44%
Ingredients, %					
Corn	58.38	58.38	58.38	60.31	63.50
Soybean meal, 44% CP	34.92	34.78	34.58	32.90	29.59
Corn gluten	3	3	3	3	3
Vegetable oil	1.11	1.11	1.11	1.11	1.11
Limestone	0.7	0.7	0.7	0.7	0.7
Dicalcium phosphate	0.97	0.97	0.97	0.97	0.97
NaCl	0.2	0.2	0.2	0.2	0.2
DL-Met	0.17	0.17	0.17	0.18	0.19
L-Lys HCL	0.30	0.44	0.64	0.38	0.49
Vitamin-Mineral Premix	0.25	0.25	0.25	0.25	0.25
Total	100	100	100	100	100
Nutritional Content					
DM, %	87.62	87.49	87.31	87.53	87.40
CP, %	22.80	22.87	22.97	22.17	21.11
CF, %	2.69	2.68	2.67	2.65	2.59
Ash, %	5.36	2.62	5.43	2.63	4.74
EE, %	3.75	3.75	3.75	3.81	3.89
ME, Kcal/g	3.03	3.04	3.04	3.05	3.08
Total AA, %					
Lys	1.44	1.54	1.68	1.44	1.44
Met	0.56	0.56	0.56	0.56	0.56
Met + cys	1.08	1.08	1.08	1.07	1.06
Thr	0.96	0.96	0.96	0.95	0.94
Val	1.08	1.08	1.08	1.07	1.06
Leu	1.58	1.58	1.58	1.56	1.55
Ileu	0.97	0.97	0.97	0.97	0.97
Arg	1.52	1.52	1.52	1.51	1.51

1. Vitamin and mineral premix: vitamin premix provided the following per kilogram of diet: vitamin A, 9000 IU; cholecalciferol, 2000 IU; vitamin E, 36 IU; vitamin K3, 2 mg; thiamine, 1.8 mg; riboflavin, 6.6 mg; pantothenic acid, 10 mg; niacin, 30 mg; choline chloride, 250 mg; biotin, 0.1 mg; folic acid, 1 mg; pyridoxine 3.0 mg; vitamin B12, 0.015 mg; BHT, 1 mg. 2. Trace mineral premix provide the following in milligrams per kilogram of diet: iron, 50 mg; zinc, 85 mg; manganese, 100 mg; iodine, 1 mg; copper, 10 mg; selenium, 0.2 mg. 3. Represents dietary electrolyte balance as defined by dietary Na + K – Cl (in mEq/kg of diet).

**Table 2:** The composition of the experimental diets and proximate chemical analysis during the growing period.

Treatments	C	Gr1	Gr2	Gr3	Gr 4
CP %	Standard protein 21.5%			20.5% CP	19.5% CP
Lysine content	1.29% Lys	1.40% (110% from basal ration)	1.53% (120% from basal ration)	1.29%	1.29%
Ingredients, %					
Corn	61.17	61.17	61.17	64.20	67.26
Soybean meal, 44% CP	31.34	31.18	31	28.5	25.3
Corn gluten	3	3	3	3	3
Vegetable oil	2	2	2	1.7	1.7
Limestone	0.7	0.7	0.7	0.7	0.7
Dicalcium phosphate	0.97	0.97	0.97	0.97	0.97
NaCl	0.2	0.2	0.2	0.2	0.2
DL-Met	0.14	0.14	0.14	0.15	0.17
L-Lys HCL	0.23	0.39	0.57	0.33	0.45
Vitamin-Mineral Premix	0.25	0.25	0.25	0.25	0.25
Total	100	100	100	100	100
Nutritional Content					
DM, %	87.74	87.59	87.43	87.59	87.45
CP, %	21.39	21.47	21.56	20.52	19.51
CF, %	2.61	2.60	2.59	2.56	2.51
Ash, %	4.55	5.08	4.74	4.32	4.7
EE, %	4.71	4.71	4.71	4.50	4.58
ME, Kcal/g	3.11	3.12	3.12	3.12	3.15
Total AA, %					
Lys	1.29	1.40	1.53	1.29	1.29
Met	0.51	0.51	0.51	0.51	0.51
Met + cys	0.99	0.99	0.99	0.97	0.96
Thr	0.88	0.88	0.88	0.87	0.86
Val	1.01	1.01	1.01	1.00	1.00
Leu	1.42	1.42	1.42	1.41	1.41
Ileu	0.89	0.89	0.89	0.87	0.86
Arg	1.37	1.37	1.37	1.36	1.35

Vitamin and mineral premix, see Table 1.

**Table 3:** The composition of the experimental diets and proximate chemical analysis during the finishing phase.

Treatments	C	Gr1	Gr2	Gr3	Gr 4
CP %	Standard protein 19.5%			18.5% CP	17.5% CP
Lysine content	1.16% Lys	1.3% (110% from basal ration)	1.41% (120% from basal ration)	1.16%	1.16%
Ingredients, %					
Corn	65.51	65.9	65.9	68.03	71.76
Soybean meal, 44% CP	26.03	25.42	25.27	23.4	20.10
Corn gluten	3	3	3	3	3
Vegetable oil	2.96	2.96	2.96	2.96	2.4
Limestone	0.7	0.7	0.7	0.7	0.7
Dicalcium phosphate	0.97	0.97	0.97	0.97	0.97
NaCl %	0.2	0.2	0.2	0.2	0.2
DL-Met	0.12	0.13	0.13	0.14	0.15
L-Lys HCL	0.26	0.47	0.62	0.35	0.47
Vitamin-Mineral Premix	0.25	0.25	0.25	0.25	0.25
Total	100	100	100	100	100
Nutritional Content					
DM, %	87.76	87.57	87.43	87.66	87.46
CP, %	19.46	19.43	19.51	18.63	17.63
CF, %	2.50	2.48	2.47	2.45	2.40
Ash, %	4.55	5.08	4.74	4.32	4.7
EE, %	5.78	5.79	5.79	5.85	5.40
ME, Kcal/g	3.22	3.22	3.22	3.24	3.24
Total AA, %					
Lys	1.16	1.30	1.41	1.16	1.16
Met	0.47	0.47	0.47	0.47	0.47
Met + cys	0.94	0.94	0.94	0.93	0.91
Thr	0.81	0.81	0.80	0.80	0.79
Val	0.92	0.91	0.91	0.90	0.90
Leu	1.31	1.31	1.31	1.30	1.30
Ileu	0.83	0.83	0.83	0.81	0.81
Arg	1.21	1.21	1.21	1.20	1.20

Vitamin and mineral premix, see Table 1.

**Table 4:** Effects of variable concentrations of dietary CP and Lys on live body weight gain (BWG), feed intake (FI), and feed conversion ratio (FCR) of broiler chickens during the trial.

Treatments	Experimental Period			
	Starter, 10–20 d	Grower, 21–30 d	Finisher, 31–42 d	Cumulative, 10–42 d
BWG, g				
C	605.7 ^b^	971.15 ^b^	1061.4 ^b^	2638.2 ^bc^
Gr1	613.7 ^b^	1028.1 ^a^	1180.9 ^a^	2822.66 ^a^
Gr2	706.9 ^a^	931.67 ^c^	1040.9 ^b^	2679.63 ^b^
Gr3	708.5 ^a^	944.33 ^bc^	986.6 ^b^	2639.39 ^bc^
Gr4	618.7 ^b^	884.67 ^d^	1045.5 ^b^	2548.82 ^c^
SEM	11.64	10.75	39.30	60.66
*p*-value	<0.001	<0.001	0.02	0.007
FI, g/bird				
C	854.00 ^b^	1011.00 ^b^	2133.00 ^b^	3998.0 ^b^
Gr1	853.00 ^b^	1051.00 ^ab^	2055.00 ^bc^	3959.0 ^b^
Gr2	933.20 ^a^	1108.69 ^a^	2686.00 ^a^	4727.9 ^a^
Gr3	857.30 ^b^	1040.00 ^b^	1944.00 ^c^	3841.3 ^b^
Gr4	841.40 b	911.21 ^c^	2164.00 ^b^	3916.6 ^b^
SEM	18.25	20.41	42.58	91.24
*p*-value	0.004	<0.001	<0.001	<0.001
FCR, g:g				
C	1.41 ^a^	1.04 ^c^	2.01 ^b^	1.52 ^b^
Gr1	1.39 ^ab^	1.02 ^c^	1.74 ^c^	1.40 ^c^
Gr2	1.32 ^c^	1.19 ^a^	2.58 ^a^	1.76 ^a^
Gr3	1.21 ^d^	1.10 ^b^	1.97 ^b^	1.46 ^bc^
Gr4	1.36 ^b^	1.03 ^c^	2.07 ^b^	1.54 ^b^
SEM	0.011	0.012	0.071	0.016
*p*-value	<0.001	<0.001	<0.001	<0.001

^a–d^ Means followed by the same letter are not significantly different at *p* < 0.05. Data are presented as mean ± SEM. C, control group; the Lys contents were increased to 10% more than recommended control basal requirements (C) in the second group (Gr1) and 20% in the third group (Gr2) with the same recommended CP% as in the C group, while the fourth group (Gr3) had a 1% lower CP content and the fifth group had a 2% lower CP content with similar Lys content to the C group.

**Table 5:** Effects of variable concentrations of dietary CP and Lys on serum protein profile of broiler chickens.

Treatments	Urea	Creatinine	Total Protein	Albumin	Globulin
C	15.50 ^a^	0.26 ^a^	2.60 ^b^	1.30	1.30 ^b^
Gr1	10.50 ^bc^	0.16 ^b^	2.70 ^ab^	1.40	1.30 ^b^
Gr2	7.00 ^c^	0.19 ^b^	2.97 ^a^	1.37	1.60 ^a^
Gr3	13.33 ^ab^	0.20 ^b^	2.70 ^ab^	1.43	1.30 ^b^
Gr4	11.33 ^b^	0.18 ^b^	2.80 ^ab^	1.50	1.30 ^b^
SEM	0.88	0.014	0.04	0.06	0.06
*p*-value	<0.001	<0.001	0.04	0.39	0.04

^a–c^ Means followed by the same letter are not significantly different at *p* < 0.05. Data are presented as mean ± SEM. For abbreviations, see Table 4.

**Table 6:** Effects of variable concentrations of dietary CP and Lys on the hepatic and renal efficiency of broiler chickens.

Treatments	ALT	AST	Total Bilirubin	Direct Bilirubin	In-Direct Bilirubin
C	7.67 ^ab^	268.00 ^c^	0.33	0.10	0.23
Gr1	7.33 ^ab^	291.67 ^b^	0.33	0.10	0.23
Gr2	6.50 ^b^	262.00 ^c^	0.33	0.10	0.23
Gr3	9.50 ^a^	355.50 ^a^	0.40	0.13	0.27
Gr4	7.33 ^ab^	273.00 ^c^	0.30	0.10	0.20
SEM	0.67	4.05	0.07	0.02	0.06
*p*-value	0.04	<0.001	0.72	0.96	0.85

^a–c^ Means followed by the same letter are not significantly different at *p* < 0.05. Data are presented as mean ± SEM. For abbreviations, see Table 4. ALT, alanine aminotransferase; AST, aspartate aminotransferase.

**Table 7:** Effects of variable concentrations of dietary CP and Lys on serum lipid profile of broiler chickens.

Treatments	Triglycerides	Cholesterol	HDL	LDL	VLDL
C	75.00	140.00	119.33 ^b^	5.67	15.00
Gr1	66.67	162.33	146.33 ^a^	7.00	13.33
Gr2	63.33	144.67	126.33 ^ab^	5.67	12.67
Gr3	73.33	153.67	132.33 ^ab^	6.67	14.67
Gr4	76.67	159.00	135.33 ^ab^	8.33	15.33
SEM	5.30	9.83	5.98	2.50	1.05
*p*-value	0.13	0.19	0.01	0.81	0.12

^a–b^ Means followed by the same letter are not significantly different at *p* < 0.05. Data are presented as mean ± SEM. For abbreviations, see Table 4. HDL, high-density lipoprotein; LDL, low-density lipoprotein; VLDL, very LDL.

## Data Availability

The data presented in this study are available on request from the corresponding author.
